# Reprocessible, Reusable, and Self-Healing Polymeric Adsorbent for Removing Perfluorinated Pollutants

**DOI:** 10.3390/ma17215170

**Published:** 2024-10-23

**Authors:** Sun Ju Kim, Minjoon Baek, Jihye Choe, Jaeman J. Shin

**Affiliations:** 1 Department of Materials Science and Engineering, Soongsil University, Seoul 06978, Republic of Korea; sju8482@soongsil.ac.kr (S.J.K.); pki77777@naver.com (M.B.); jihea798@naver.com (J.C.); 2 Department of Green Chemistry and Materials Engineering, Soongsil University, Seoul 06978, Republic of Korea

**Keywords:** fluorinated hydrogel, adsorbent, reprocessible, reusable, self-healing

## Abstract

Here, we report a reprocessible, reusable, self-healing, and form-switching polymeric adsorbent for remediating fluorinated pollutants in water. The copolymer hydrogel is designed to contain fluorophilic segments and cationic segments to induce strong binding with perfluorinated pollutants. The sorption performance reveals rapid and quantitative removal of these pollutants, driven by the synergistic effect of fluorophilic and electrostatic interaction. Importantly, a disulfide-containing dynamic crosslinker plays a crucial role in imparting multifunctionality. This enables self-healing by the restoration of crosslinks at the cut surfaces by disulfide exchange reactions and allows for the repeated use of the adsorbent via multiple adsorption–desorption cycles. Furthermore, the adsorbent is reprocessible by cleaving the crosslinks to afford linear copolymers, which can be repolymerized into a hydrogel network on demand. Also, form-switching capability is showcased through the aqueous self-assembly of linear copolymers into a fluorinated micelle, serving as another form of adsorbent for pollutant removal.

## 1. Introduction

Per- and polyfluorinated alkyl substances (PFASs) are fluorinated synthetic chemicals widely used for industrial applications and have produced numerous commercial products [1,2,3]. However, their exceptional environmental persistence has turned them into bioaccumulative pollutants, linked to several health effects in humans, including liver damage, cancer, and thyroid disease [4,5,6,7]. As a result, PFAS contamination in drinking water has become a significant public health concern, particularly for anionic long-chain PFASs such as perfluorooctanoic acid (PFOA) and perfluorooctanesulfonic acid (PFOS) [8,9]. As the environmental persistence of PFASs originates from the exceptional stability of C-F bonds, efforts to degrade PFASs via bond scission have been challenging. These attempts include oxidative [10], photochemical [11], sonochemical [12], and electrochemical degradation approaches [13,14,15], but such processes are either expensive or produce toxic byproducts [16]. Accordingly, the removal of PFASs by adsorption received attention [17], with granular activated carbon (GAC) as a leading adsorbent used on a large scale for the efficient capture and removal of PFASs to remediate contaminated water sources. Also, electrostatic adsorption using a charged solid surface is a promising method for capturing PFASs [18,19,20].

Recently, polymeric adsorbents have received great attention as they exhibit fast adsorption kinetics and high removal capacity [21]. Such high performance originates from specific interactions between the adsorbent and PFASs, including hydrogen bonding, fluorine–fluorine, and electrostatic interaction. Accordingly, various forms of polymeric adsorbents have been developed such as porous organic polymer adsorbents [22,23,24,25], hydrogel/fluorogel networks [26,27,28], micelle-embedded hydrogels [29], micro/nanogels [30,31], and block copolymer micelles [32,33]. However, efforts to integrate multiple functionalities, such as recoverability, reusability, and durability, into the polymeric adsorbents have been less investigated [34]. The recent advances in dynamic covalent chemistry show that introducing dynamic bonds facilitates the rational design of smart soft materials with adaptability, repairability, recyclability, and degradability via the rearrangement of polymeric networks in response to stimuli [35,36].

In this work, we introduce a multifunctional fluorinated copolymer hydrogel designed as a polymeric adsorbent for the efficient removal of perfluorinated contaminants in water. The hydrogel exhibits key features such as reprocessibility, reusability, self-healing capability, and form-switching ability. The copolymer hydrogel was designed to include segments with fluorophilic group, cationic quaternary ammonium group, hydrophilic segments, and dynamic disulfide-containing crosslinker. Using PFOA as a model contaminant, the hydrogel demonstrated excellent sorption performance, achieving rapid and quantitative removal of PFOA across a range of concentrations in aqueous solutions. This result is attributed to the combination of fluorophilic and quaternary ammonium groups, which facilitate favorable interaction with PFOA via fluorophilic and electrostatic interactions. Importantly, disulfide-containing dynamic crosslinkers provide self-healing capabilities, reusability, reprocessibility, and form switchability. The durability of the hydrogel was confirmed by its self-healing capability, enabled by the re-formation of disulfide crosslinks at the cut surfaces. Also, the hydrogel was reusable, as evidenced by multiple adsorption–desorption cycles. Reprocessibility was demonstrated by cleaving the disulfide crosslinks, yielding linear random copolymers that can be dissolved in organic solvents. These copolymers were then easily repolymerized by re-forming disulfide crosslinks, thus regenerating a hydrogel network. Additionally, the bulky hydrogel was transformed into fluorinated micelles via the aqueous self-assembly of linear random copolymers, serving as another form of polymeric adsorbent used for the removal of PFOA.

## 2. Materials and Methods

### 2.1. Materials

Poly(ethylene glycol) methyl ether acrylate (PEGA, *M_n_* = 484 g mol^−1^), poly(ethylene glycol) dimethacrylate (PEGdiMA, *M_n_* = 550 g/mol), 2-(dimethylamino) ethyl methacrylate (DMAEMA, 98%), 2,2-dimethoxy-2-phenylacetophenone (DMPA, 99%), deuterated water (D_2_O, 99% atom % D), perfluorooctanoic acid (PFOA, 95%), and *D*,*L*-dithiothreitol (DTT, 97%), dialysis tubing with a molecular weight cutoff (MWCO) of 2000 were purchased from Sigma-Aldrich (St. Louis, MO, USA). Trifluoroethanol (TFE), *1H*, *1H*, *5H*-octafluoropentyl methacrylate (OFPMA, stabilized with MEHQ, 98%), and *N*, *N’*-bis(acryloyl)cysteamine (BAC, 95%) were purchased from TCI. All other commercial solvents were obtained from Samchun Chemical and used as received (Seoul, Republic of Korea). All monomers were purified by passing through the basic alumina plug before use.

### 2.2. Synthesis of Fluorinated Copolymer Hydrogel

Fluorinated hydrogels were synthesized by photoinitiated radical copolymerization using DMPA as a photoinitiator. The desired amounts of monomers including PEGA, DMAEMA, OFPMA, and DMPA were added to a 10 mL vial. BAC was dissolved in ethanol/tetrahydrofuran (EtOH/THF) and added to the monomer solution. After obtaining a fully dissolved homogeneous solution, polymerization was performed by irradiating UV light using a commercially available UV nail lamp (λ = 365 nm, 36 W). The solution gelled after 2 h, affording disk-shaped hydrogel. Unreacted water-soluble monomers (PEGA and DMAEMA) were removed by repeatedly washing the hydrogels by adding DI water into the reaction vial to fully soak the hydrogel, perform sonication for 5 min, and discard the liquids. After repeating this process at least 5 times, the hydrogel was dried in a vacuum for 1 day. Note that the OFPMA monomer was removed during this process. If residual monomers were observed in FT-IR, the whole purification process was repeated. The composition of DMAEMA, OFPMA, and PEGA varied with respect to the total mole percent (100% = 4.0 mmol), while the feed percentage of the BAC crosslinker was fixed at 5%. The quaternization of the DMAEMA group was performed by soaking 25 mg of hydrogel in 3 mL of EtOH solution containing 0.1 mL of methyl iodide (CH_3_I) at room temperature for 1 day. The conversion of the synthesized hydrogel was calculated using the following equation:

$$\%\;conversion=\frac{Total\;weight\;of\;hydrogel\;after\;drying}{Total\;weight\;of\;the\;added\;monomer}\times 100$$

where the weight of the hydrogel after drying was determined until reaching a constant weight.

### 2.3. General Procedure for Adsorption and Desorption of PFOA

The sorption performance of the fluorinated hydrogels was investigated by using PFOA as a model pollutant. Briefly, 25 mg of hydrogel was placed in a 5 mL vial equipped with a magnetic stirring bar. To this vial, 1 mL of PFOA solution (*C*_0_ = 25, 50, 75, 100, 200 mg/L of PFOA in EtOH/D_2_O (1/1 = *v*/*v* (%)) was added. The mixture was stirred at 80 rpm for 10 h at room temperature. After adsorption, the liquids were transferred to another container to separate the swollen hydrogel. By measuring the residual amount of PFOA in the separated aqueous liquid, the concentration of PFOA residue was calculated. In detail, a known amount of TFE, as an internal standard, was added to the separated aqueous liquid followed by characterization using ^19^F NMR (Figure S1). Comparing the integration ratio between the characteristic peaks from TFE (−76.0 ppm) and PFOA (−81.5 ppm) revealed the residual amount of PFOA in the liquid. Using this value, the removal efficiency of PFOA was calculated using the following equation:

$$\%\;PFOA\;removal=\frac{\left(C_{0}-C_{r}\right)}{C_{0}}\times 100$$

where *C*_0_ is the initial concentration of PFOA, and *C_r_* is the residual concentration of PFOA in the aqueous solution after adsorption. Measurements were performed more than 3 times, and an average value of % PFOA removal is reported.

The adsorption capacity of PFOA, defined as the mass of adsorbed PFOA per the mass of the adsorbent, was determined using the following equation:

$$Adsorption\;capacity=\frac{\left(C_{0}-C_{r}\right)V}{m}$$

where *C*_0_ is the initial concentration of PFOA, and *C_r_* is the residual concentration of PFOA in the solution after adsorption. *V* is the solution volume in liters, and *m* is the mass of the fully dried hydrogel. Measurements were performed more than 3 times, and the results are reported as an average value.

For the desorption of PFOA, methanol was used as a PFOA-extracting solvent due to its superior solubility of PFOA compared to water. The desorption of PFOA was performed by soaking the PFOA-adsorbed hydrogel in methanol, followed by sonication for 5 min and disposal of the methanol solution. This process was repeated 5 times, followed by drying in a vacuum for 24 h. The reusability of the hydrogel was examined by conducting three runs of PFOA adsorption–desorption procedures.

### 2.4. Self-Healing Experiments

The self-healing behavior of the copolymer hydrogel was examined by cutting the disk-shaped hydrogel into two pieces using a razor blade. After bringing them back into contact, the material was placed inside a vacuum oven at 80 °C for a given time. The durability of the healed materials was compared to the pristine samples by manually stretching the hydrogel.

### 2.5. Cleavage of Disulfide Crosslinks and Reprocessing of the Hydrogels

Disulfide bonds were cleaved by placing a hydrogel in a THF solution containing an excess amount of DTT (20 mg/mL) at 70 °C under stirring. The cleavage of crosslinks was notable as the undissolved, crosslinked hydrogel became fully dissolved as a solution after a certain period of reaction time. For hydrogel reprocessing experiments, this solution was concentrated two-fold and was directly exposed to the UV light for 2 h. The gelled product indicates the re-formation of disulfide crosslinks.

### 2.6. Formation of Fluorinated Micelles and Their Adsorption

For generating fluorinated micelles, 1 mL of the DI water was added dropwise at a rate of 1 mL/h using a syringe pump into the 1 mL of DTT-treated hydrogel solution under stirring at 300 rpm. When the addition of DI water was complete, the turbid solution was observed, and additional stirring for 1 h was performed to ensure the generated micelles reached equilibrium. The turbid solution was subjected to dialysis with water for 1 day using a dialysis tubing with a MWCO of 2000, in order to exchange the surrounding solution to water.

The adsorption capability of the fluorinated micelles was examined by adding 1 mL of PFOA solution (*C*_0_ = 25, 50, 75, 100, 200 mg/L of PFOA in D_2_O) to a 1 mL of aqueous solution containing micelles and stirring for 10 h at room temperature. Afterward, the solution was subjected to dialysis with water using 2000 MWCO dialysis tubing, and the resulting aqueous solution was analyzed to obtain *C_r_*.

### 2.7. Characterization

Attenuated total reflection Fourier transform infrared spectroscopy (ATR FT-IR) was performed using a VERTEX 70 FT-IR spectrometer (Bruker, Billerica, MA, USA). Nuclear magnetic resonance (^19^F and ^1^H NMR) spectra were recorded on a JEOL 500 NMR spectrometer. For ^1^H NMR, chemical shifts (δ) were reported in ppm relative to 2.5 ppm in deuterated dimethyl sulfoxide (DMSO-d_6_) and 4.65 ppm for deuterated water (D_2_O). Size exclusion chromatography (SEC) was conducted on a Waters GPC system, equipped with a Waters 1515 pump and Waters 2414 refractive index detector using THF as a mobile phase with a flow rate of 1.0 mL/min. Number-average molar weight (*M*_w_) and mass-average molar mass (*M*_m_) were determined relative to linear polystyrene standards and used to estimate the molar mass dispersity (*Đ* = *M*_w_/*M*_n_). Dynamic light scattering (DLS) measurements were conducted using Malvern Zetasizer to determine the diameter of the particles. Transmission electron microscopy (TEM) (LIBRA 120, accelerating voltage of 120 kV, Carl Zeiss, Jena, Germany) measurements were performed to characterize the size of the micelles. The samples were prepared by drop-casting the dilute solution containing micelles onto Cu grids coated with a 20 nm thick carbon film.

## 3. Results and Discussion

### 3.1. Synthesis of Fluorinated Hydrogel Adsorbent

**Scheme 1:**
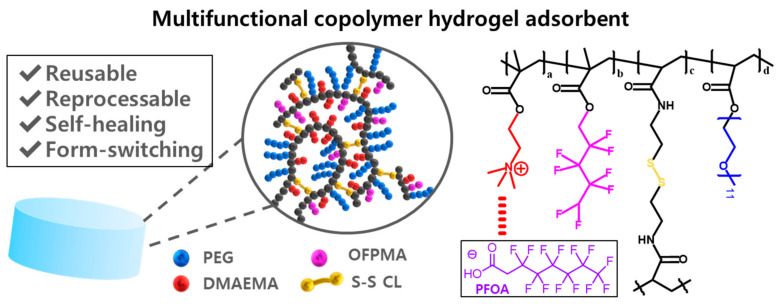
Illustration and chemical structure of the fluorinated hydrogel adsorbent.

Scheme 1 illustrates the fluorinated hydrogel developed in this work and its chemical structure. The hydrogel with a branched linear structure was designed to contain OFPMA, DMAEMA, and PEGA as a repeating unit and a disulfide-containing crosslinker. OFPMA serves as a fluorophilic sorption site for the capture of PFOA, and the tertiary amine group in DMAEMA can be quaternized to attract anionic group of PFOA pollutants via electrostatic interaction. These two components are expected to synergistically leverage the fluorophilic and electrostatic interaction to exhibit high performance in PFOA removal. PEGA constitutes the majority of the remaining components as a hydrophilic segment, providing stability in the water. Importantly, the key component in our work is the dynamic crosslinker that can cleave and re-form in response to a stimulus, as indicated by the recent advances in dynamic covalent chemistry that allow for designing materials with adaptability, repairability, recyclability, and degradability via the rearrangement of polymeric networks [35,36]. We employed disulfides as a dynamic bond due to their robustness and dynamicity. The robustness stems from ca. 60 kcal mol^−1^ of bond dissociation energy, and the dynamicity in the reversible linkages originates from the fast thiol–disulfide exchange reactions under mild conditions [37,38]. The BAC crosslinker used in this work is a simple and commercially available disulfide-containing crosslinker that finds widespread use in building multi-responsive micro/nanogels for delivery systems [39,40,41]. Overall, the presence of the dynamic crosslinker in the fluorinated copolymer is expected to provide reprocessibility, reusability, self-healing, and degradability to the polymeric adsorbent.

The fluorinated hydrogel was synthesized through photoinitiated radical copolymerization of DMAEMA, OFPMA, BAC, and PEGA, followed by the quaternization of the tertiary amine groups by treating the copolymer with methyl iodide (Scheme S1). Table 1 summarizes the composition and molecular weight of all the samples used in this work. The sample code is AXFY, where A and F represent the molar feed percentage of DMAEMA and OFPMA, respectively, and the plus sign indicates samples with quaternized dimethylamino groups. The feed percentage of each monomer was calculated relative to the total mole of the hydrogel while maintaining a constant crosslinker (BAC) concentration of 5%. A high conversion of monomers into hydrogels, ranging from 82 to 92%, confirmed successful polymerization. Also, the characterization of the number-average molecular weight (M_n_) by GPC showed similar values ranging from 500 to 532 kg/mol for all samples (Figure S2), consistent with the high conversion and the targeted monomer-to-initiator ratio. Furthermore, the actual composition of the synthesized hydrogel was characterized by ^1^H NMR, by comparing the integration of the characteristic peaks and calculating the number of repeating units of each monomer (Figure S3). The actual composition of hydrogels was similar to that of feed percentages of each monomer, reflecting no sign of compositional drift.

**Figure 1:**
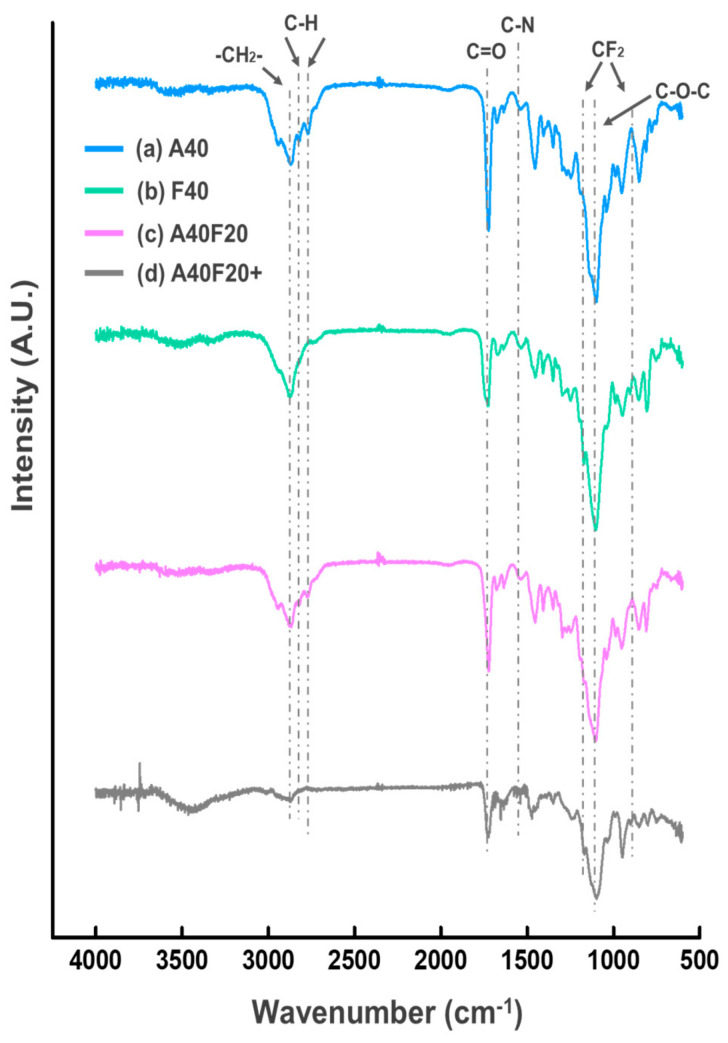
FT-IR overlay of (a) A40, (b) F40, (c) A40F20, and (d) A40F20+.

Further structural characterization of hydrogels was performed by ATR-FT-IR (Figure 1). The characteristic peaks associated with key functional groups were identified. For example, the peaks at 2750 and 2820 cm^−1^ in the A40 sample are associated with the C-H stretch from the -N(CH_3_)_2_ in DMAEMA (Figure 1a). The peaks at 900 and 1041 cm^−1^ of F40 in Figure 1b are attributed to the C-F_2_ bond, supporting the presence of OFPMA groups. Evidently, A40F20 and A40F20+ contain both peaks associated with -N(CH3)_2_ and C-F_2_, reflecting the presence of both DMAEMA and OFPMA (Figure 1c,d). The common features observed for all samples are the peaks at 1100, 1722, and 2867 cm^−1^, corresponding to C-O-C, C=O, and -CH_2_-, groups arising from PEG and matrix and (meth)acrylic backbones. Also, the C-N stretch peak at 1539 cm^−1^ reflects that the BAS crosslinker is successfully contained in the hydrogels. Additionally, the absence of the C=C stretching vibration at 1639 cm^−1^ for all samples indicates the complete removal of residual methacrylic monomers. Additional FT-IR characterization details are provided in Figures S4–S7.

### 3.2. Sorption Performance

**Figure 2:**
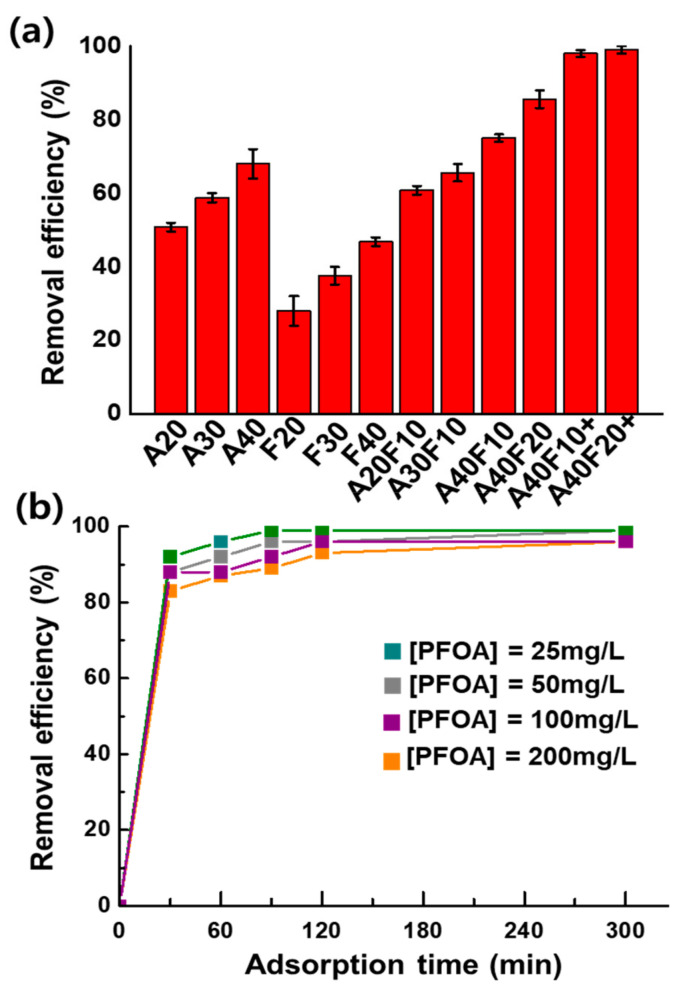
(**a**) Removal efficiency of PFOA using different compositions of hydrogels. The initial concentration of PFOA was fixed at 50 mg/L; (**b**) adsorption kinetics of PFOA using A40F20+ hydrogel at different concentrations.

The sorption performance of fluorinated hydrogels was investigated using PFOA as a model pollutant, a long-chain anionic fluorinated surfactant abundantly used in the industry [42,43]. Removal efficiencies were screened by examining samples with varying compositions (Figure 2a). Hydrogels with only fluorous groups (F20, F30, and F40) showed low removal efficiency below 40%. Hydrogels with only DMAEMA (A20, A30, and A40) afforded slightly improved removal efficiencies ranging from 50 to 70%, indicating that the favorable interaction of the dimethylamine group with PFOA is greater than the fluorophilic interaction between OFPMA and PFOA. The A40F20 hydrogel, containing both DMAEMA and OFPMA, showed superior removal efficiency, reaching 86% at a PFOA concentration of 25 mg/L. This result reflects that the synergistic interaction of OFPMA and DMAEMA with PFOA contributes to improved PFOA-capturing performance. Notably, the quaternization of DMAEMA led to a dramatic improvement in removal efficiency, with 98 and 99% for A40F10+ and A40F20+, respectively. Such enhancement is attributed to the electrostatic interaction between the anionic PFOA with the quaternized ammonium group in the hydrogels. These results are consistent with the previous reports showing that fluorine−fluorine interactions between the perfluoropolyether segment of the sorbent and the fluorinated tail of PFOA contribute to the recognition of PFOA molecules via fluorophilicity, and electrostatic attraction between the cationic group and the anionic headgroup of PFOA facilitates the tight binding of PFOA to suppress exchange between bound and unbound PFOA molecules [33,44]. Additionally, the adsorption capability of the hydrogel without disulfide crosslinker (A40F20-X) was investigated as a control experiment, showing a removal efficiency of ca. 76%, comparable to that of A40F20 (80%) (Figure S8). This result indicates that the crosslinker chemistry has minimal effect on the sorption capability and thus disulfide-containing crosslinker can be readily incorporated into the hydrogel without deteriorating the sorption performance. PFOA adsorption using neat PEG hydrogel was evidently not effective, showing a removal efficiency of only 8% (Figure S8).

To understand the kinetics of adsorption, the percent removal of PFOA was analyzed as a function of time using A40F20+ as an adsorbent (Figure 2b). The majority of PFOA (ca. 82%) was rapidly adsorbed within 30 min at a high concentration of PFOA (200 mg/L). The quantitative removal of PFOA was reached after 2 h, and the adsorption of PFOA was stable with no sign of desorption observed until 20 h (Figure S9). The adsorption kinetics of A40F20+ at lower concentrations of PFOA showed a similar trend. The fast adsorption of PFOA was achieved within 30 min, where the removal efficiencies reached 88, 88, and 92% for concentrations of 25, 50, and 100 mg/L, respectively. Also, the removal efficiency reached quantitative adsorption at 2 h, and no desorption was observed over time. The maximum adsorption capacity of the adsorbents was estimated based on the sorption performance, ranging from 2.4 to 4.0 mg/g (Table S1). Such high adsorption capacity at a high concentration (200 mg/L) of PFOA in aqueous solution indicates the suitability of the hydrogel adsorbents in this work for treating water sources heavily contaminated by PFASs (c.f. environmentally relevant concentration of PFASs = 1 μg/L).

### 3.3. Reusability, Self-Healing, and Reprocessibility of the Hydrogel

**Figure 3:**
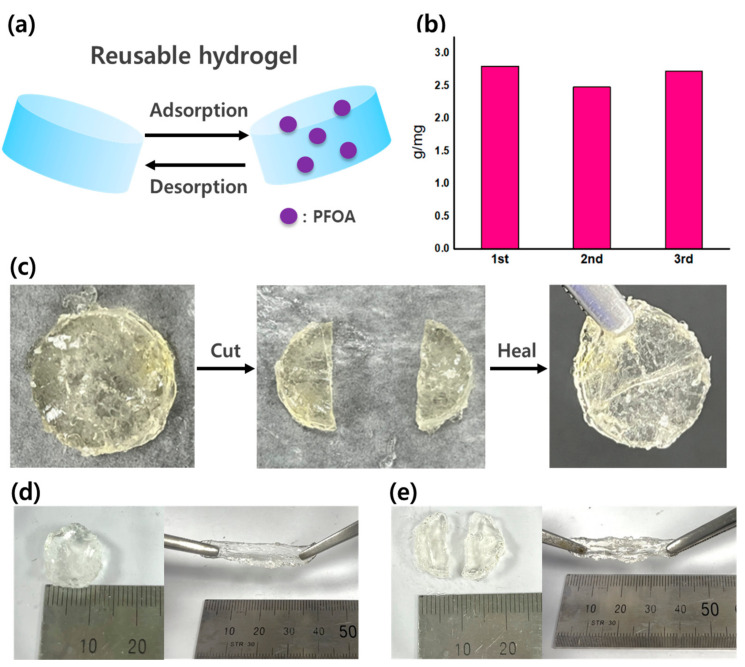
(**a**) Illustration showing the reusability of the hydrogels by repeated adsorption–desorption of PFOA; (**b**) the adsorption capacity of the hydrogel after recycling the hydrogel; (**c**) the self-healing ability of the hydrogels; stretchability (**d**) before and (**e**) after self-healing.

The reusability of the fluorinated hydrogel adsorbents is critical for real-world applications by preventing secondary pollution and increasing cost-effectiveness. Therefore, the reusability of the hydrogel was examined by estimating the adsorption capacity during a repeated adsorption–desorption of PFOA. Desorption was performed by extracting adsorbed PFOA by thoroughly washing the PFOA-swollen hydrogels with organic solvents to be employed for the next adsorption run (Figure 3a). Importantly, the adsorption capacity of the hydrogels showed little difference during the adsorption–desorption cycles with estimated adsorption capacity of 2.8, 2.5, and 2.7 mg/g for each run, respectively (Figure 3b).

Moreover, the durability of the adsorbent is important for the long-term use of materials. Benefitting from the presence of a dynamic crosslinker, the self-healing capability was realized based on the disulfide exchange reaction [45,46,47,48,49]. The disk-shaped original hydrogel was cut into two pieces and brought into contact. Afterward, heating at 80 °C accelerated a thermally activated disulfide exchange reaction, and the hydrogel self-healed within 5 min (Figure 3c). For radical-mediated disulfide metathesis, the cleavage of the disulfide bond leads to the formation of sulfur-centered radicals that can rapidly attack another disulfide bond [50,51]. Heating greatly accelerates the re-formation of disulfide bonds so that damaged materials can be quickly repaired. In addition, the stretchability of the hydrogel was retained before (Figure 3d) and after (Figure 3e) self-healing, withstanding up to 266% of its original length. The ability to self-heal in a short time, in addition to the high stretchability, reflects the structural integrity of the adsorbent materials.

**Figure 4:**
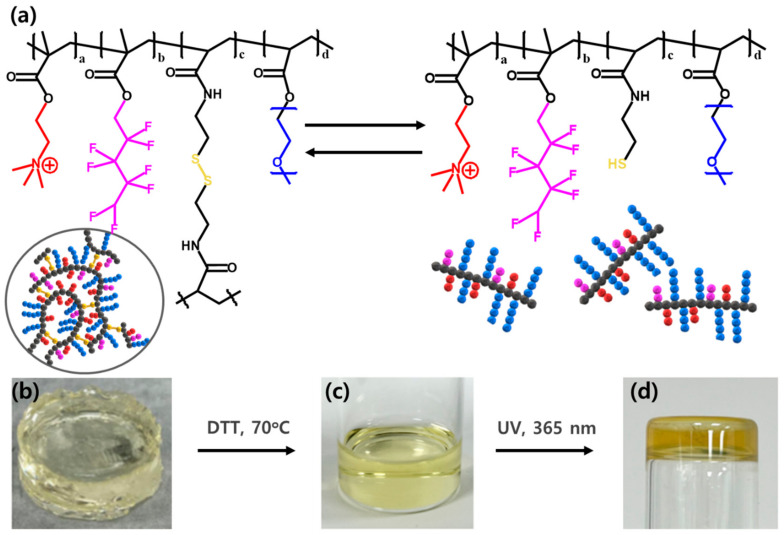
(**a**) Chemical structure showing the reduction in disulfide crosslinks and re-formation of disulfide bridge; (**b**–**d**) hydrogel reprocessing experiments: (**b**) pristine hydrogel; (**c**) solubilized hydrogel in THF after DTT treatment; (**d**) reprocessed hydrogel product after UV exposure.

Owing to the labile nature of the BAC crosslinks, we assumed that these hydrogels could be transformed into linear polymer chains and reprocessed on demand (Figure 4a). Therefore, DTT, a redox agent for cleaving disulfide bonds [52,53], was introduced to the solution soaked with A40F20 hydrogel (Figure 4b). After an hour, the insoluble gel fraction completely disappeared, thus yielding a yellowish transparent solution, indicating the cleavage of the disulfide crosslinks to be dissolved in THF (Figure 4c). Further FT-IR analysis showed that -SH peak at 2500 cm^−1^ appeared after DTT treatment due to the reduction in disulfide bonds, whereas no signal associated with -SH was found for the pristine hydrogel (Figure S10). As newly formed thiols are highly reactive toward forming another disulfide bond, exposing the solution to the UV light effectively afforded reprocessed, gelled product, as shown by the vial inversion test (Figure 4d) [54,55]. Importantly, the sorption capability of the materials showed small differences after reprocessing: the adsorption capacity of the pristine hydrogel was 2.4 mg/g, while that of the material after the reprocessing was 2.1 mg/g (Figure S11). These results showcase the reprocessibility of the adsorbent while maintaining its sorption performance.

**Figure 5:**
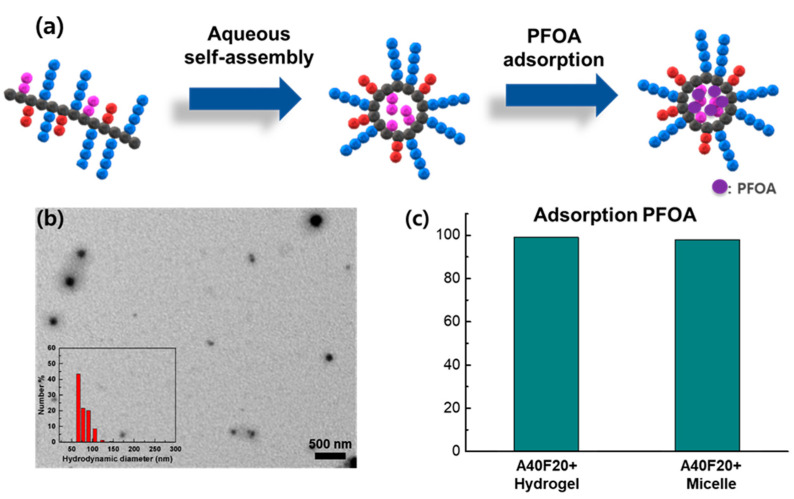
(**a**) Illustration showing the aqueous self-assembly of random copolymers into micelles and their adsorption of PFOA; (**b**) DLS (inset) and TEM image of micelles; (**c**) removal efficiency of PFOA for bulky hydrogels and micelles.

After the cleavage of BAC crosslinkers, hydrogels transform into amphiphilic random copolymers that are capable of aqueous self-assembly into fluorinated micelles, which can act as another form of polymeric adsorbent for removing PFOA in the aqueous solution (Figure 5a) [56,57,58]. Therefore, the DTT-treated solution was analyzed to confirm that the obtained material is a poly(OFPMA-*r*-DMAEMA-*r*-MEAA-*r*-PEGA) random copolymer (PMEAA: poly(*N*-2-mercaptoethyl)acrylamide)). The ^1^H NMR analysis of the product showed characteristic peaks associated with the protons arising from (meth)acrylic backbones, PEG, and the dimethyl group in PDAEMA (Figure S12a), and OFPMA groups were identified by ^19^F NMR (Figure S12b). Using this product, fluorinated micelles were generated through nanoprecipitation through the dropwise addition of DI water into the THF solution containing random copolymers under continuous stirring. Micelles were formed by the aggregation of the OFPMA groups and methacrylic backbones constituting the core, while PEG and quaternized ammonium groups were exposed to the corona to favorably interact with the aqueous surroundings, as confirmed by the NMR analysis of the micelles dispersed in D_2_O showing the disappearance of fluorine signals in ^19^F NMR (Figure S13). The resulting particle showed a hydrodynamic diameter of 78.2 ± 14.6 nm in DLS, and a similar diameter of 83.3 ± 24.5 nm was confirmed in TEM (Figure 5b). Importantly, the sorption capability of obtained micelles was analyzed by dispersing the particles in an aqueous solution containing PFOA (200 mg/L). The removal efficiency of PFOA was quantitative (99%), comparable to that of the A40F20+ bulky hydrogel before disulfide cleavage (Figure 5c). This result demonstrates that the form of the polymeric adsorbent can be effectively switched from a hydrogel type to fluorinated micelles upon the cleavage of dynamic crosslinkers followed by aqueous self-assembly while maintaining a comparable removal efficiency for PFAS pollutants.

## 4. Conclusions

In conclusion, we have demonstrated a fluorinated copolymer hydrogel as a polymeric adsorbent that can effectively remove PFASs and show multifunctionality, including reusability, self-healability, reprocessibility, and form-switching capability from bulky hydrogel into micelles. The key component for exhibiting such multifunctionality was a disulfide-based dynamic crosslinker that can undergo an exchange reaction in response to various stimuli. Fluorinated hydrogel exhibited rapid and quantitative removal of PFOA contaminants at high concentrations of PFOA, which is attributed to the synergistic effect of fluorophilic interaction and electrostatic attraction originating from the OFPMA and quaternized DMAEMA repeating units, respectively. The hydrogel was reusable multiple times via repeated adsorption–desorption of PFOA and showed self-healing capability, making it potentially durable to mechanical stresses during the remediation of a contaminated water source. Moreover, the adsorbent was reprocessible, demonstrated by the cleavage of disulfide crosslinks followed by re-crosslinking of thiolated random copolymers to yield a remolded hydrogel. Similarly, the form-switching capability of the adsorbent was realized, where the bulky hydrogel was transformed into micelles in the aqueous phase, which showed equally effective performance in terms of PFOA remediation.

## Figures and Tables

**Table 1:** Composition of fluorinated hydrogels.

Sample Code	^a^ Feed Ratio (%)	^b^ Composition (%)	Conversion (%)
A20	20:0:80	22:0:78	82
A30	30:0:70	29:0:71	88
A40	40:0:60	38:0:62	86
F20	0:20:80	0:18:82	88
F30	0:30:70	0:27:73	86
F40	0:40:60	0:43:57	92
A20F10	20:10:70	20:13:67	86
A30F10	30:10:60	31:10:59	89
A40F10	40:10:50	39:10:51	85
A40F20	40:20:40	41:20:39	82
A40F10+	40:10:50	39:10:51	N.A. ^d^
A40F20+	40:20:40	41:20:39	N.A. ^d^
A40F20-X ^c^	40:20:40	38:18:44	86
A0F0-X ^c^	0:0:100	0:0:100	92

^a, b^ [DMAEMA]:[OFPMA]:[PEGA] or a:b:d. ^c^ X indicates a control hydrogel with PEGdiMA as a non-reactive crosslinker instead of a dynamic crosslinker BAC. ^d^ Not applicable.

## Data Availability

The original contributions presented in the study are included in the article and Supplementary Materials, further inquiries can be directed to the corresponding author.

## Supplementary Materials

The following supporting information can be downloaded at: https://www.mdpi.com/article/10.3390/ma17215170/s1, Figure S1: 19F NMR spectra showing the residual PFOA in the aqueous solution. Scheme S1: Full synthesis scheme of the hydrogels. Figure S2: GPC results for the selected hydrogels. Figure S3: 1H NMR results for the hydrogels. Figure S4: FT-IR spectra for A20-A40. Figure S5: FT-IR spectra for F20-F40. Figure S6: FT-IR spectra for A20F10, A30F10, A40F10, A40F20. Figure S7: FT-IR spectra for A40F10+. Figure S8: Removal efficiency of A40F20-X and A0F0-X. Figure S9: Adsorption kinetics of PFOA using A40F20+ hydrogel at 100 mg/L. Table S1: Summary of estimated adsorption capacity. Figure S10: FT-IR spectra before and after DTT treatment. Figure S11: Adsorption capacity of pristine and reprocessed hydrogel. Figure S12: 1H and 19F NMR spectra after cleavage of disulfide bond. Figure S13: 19F NMR of the micelles in D2O.
