# Supplementary material for: Reprocessible, Reusable, and Self-Healing Polymeric Adsorbent for Removing Perfluorinated Pollutants
Source: Materials (Basel). 2024 Oct 23;17(21):5170. doi: 10.3390/ma17215170 (PMC11547204; doi:10.3390/ma17215170)
Supplement: Supplementary file 1 [file materials-17-05170-s001.zip › materials-3258063-supplementary.pdf]

Article

Reprocessible, Reusable, and Self-Healing Polymeric Adsorbent for Removing Perfluorinated Pollutants

Sun Ju Kim [1], Minjoon Baek [1], Jihye Choe [1,2] and Jaeman J. Shin [1,2,*]

[1] Department of Materials Science and Engineering, Soongsil University, Seoul 06978, Republic of Korea
[2] Department of Green Chemistry and Materials Engineering, Soongsil University, Seoul 06978, Republic of Korea
* Correspondence: jshin@ssu.ac.kr

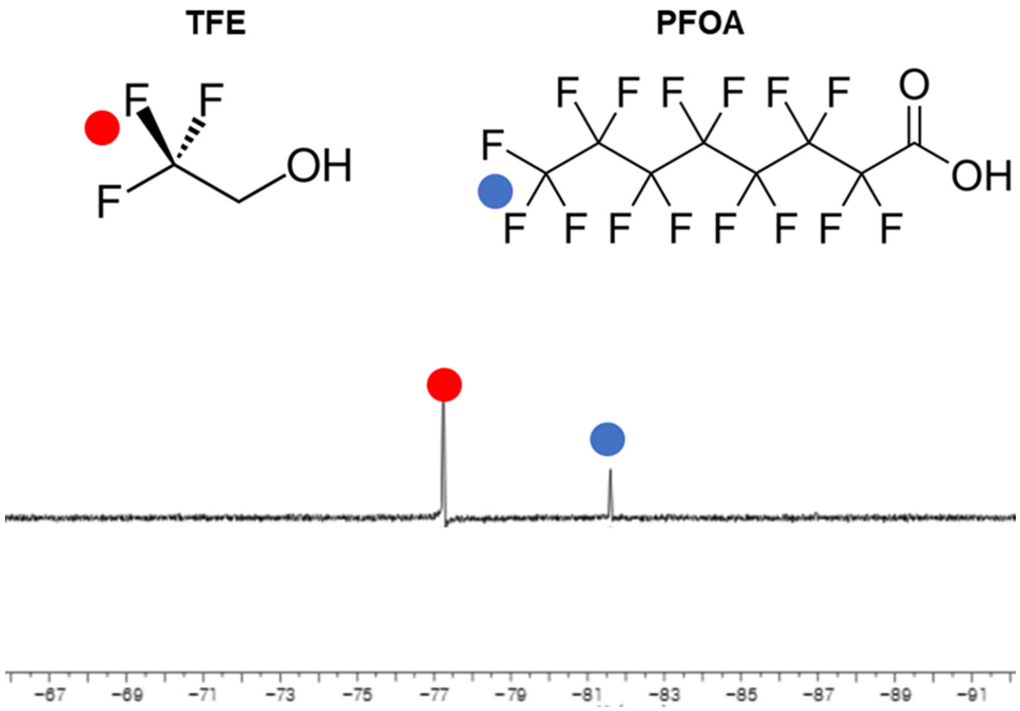

Figure S1. ^{19}F NMR spectra showing the residual PFOA in the aqueous solution with TFE as an internal standard. $-CF_3$ in TFE -76.0 ppm was referenced to the signal from $-CF_3$ in PFOA at -81.5 ppm to calculate the concentration of the residual amount of PFOA (C_r) after adsorption experiments.

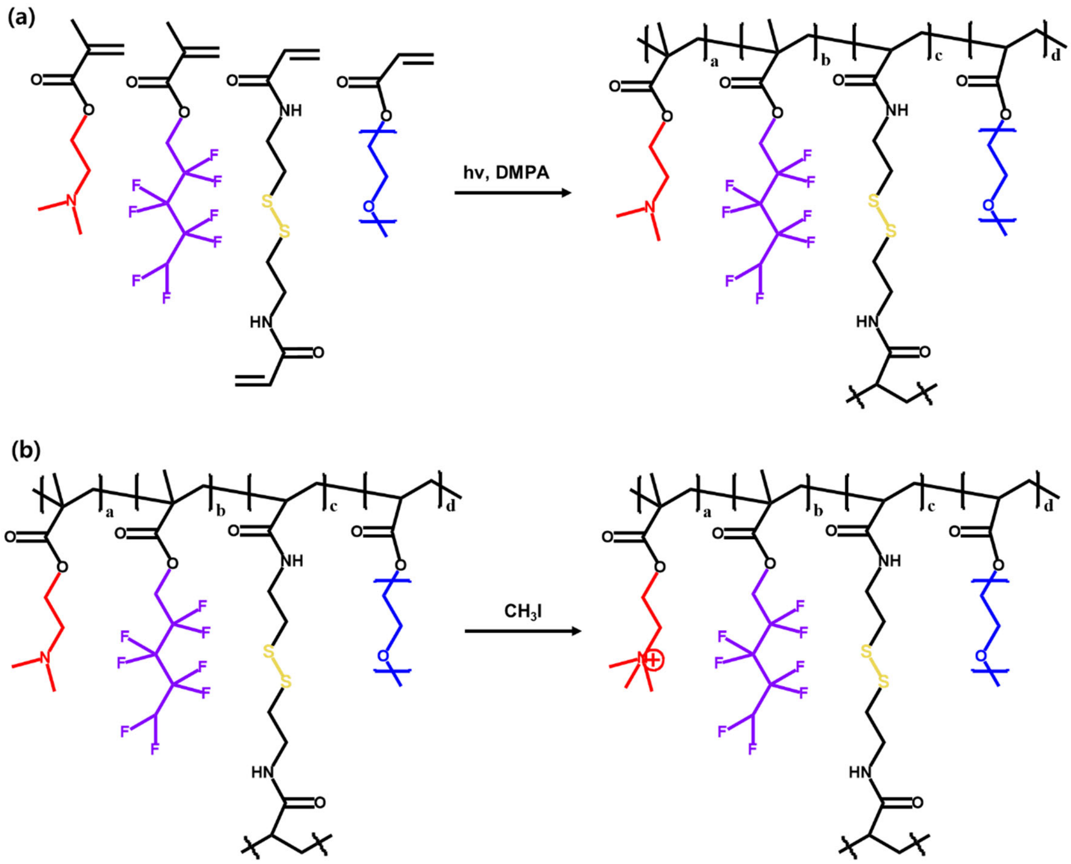

Scheme S1. Scheme for the (a) synthesis of the fluorinated copolymer hydrogel by photoinitiated free-radical polymerization followed by (b) quaternization of tertiary amine groups.

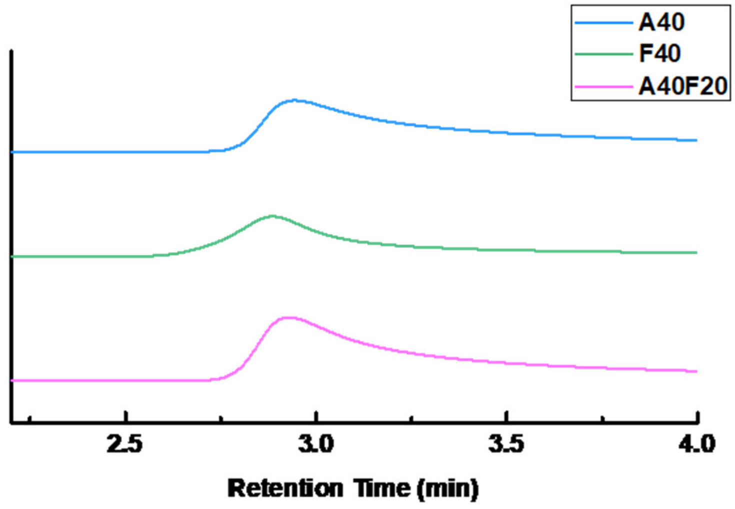

Figure S2. GPC results for the selected, representative hydrogels in Table 1.

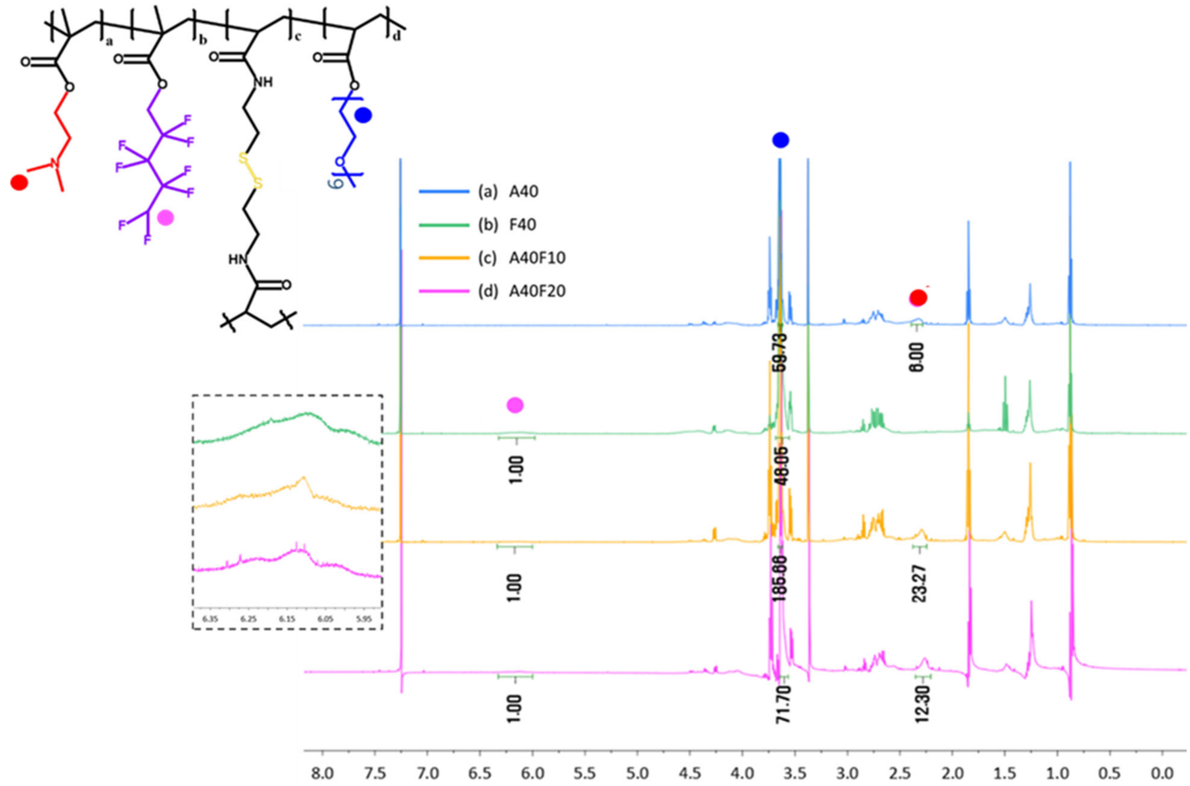

Figure S3. ^{1}H NMR results of hydrogels. Signals from protons of -$N(CH_3)_2$ in PDMAEMA, PEG, and -CF_2H in POFPMA appear in 2.3 ppm (pink), 3.5 ppm (blue), and 6.2 ppm (red), respectively.

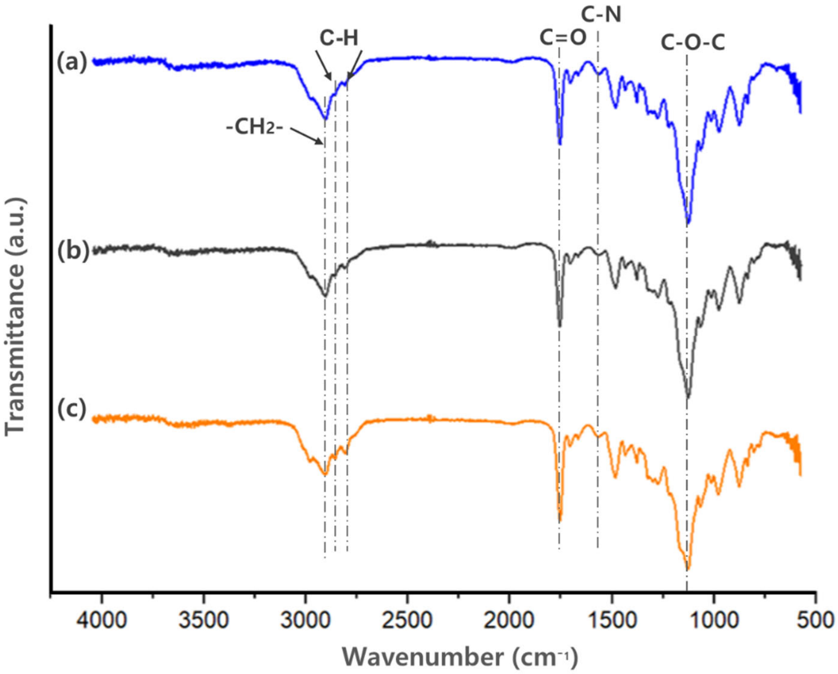

Figure S4. FT-IR spectra of (a) A20, (b) A30, and (c) A40.

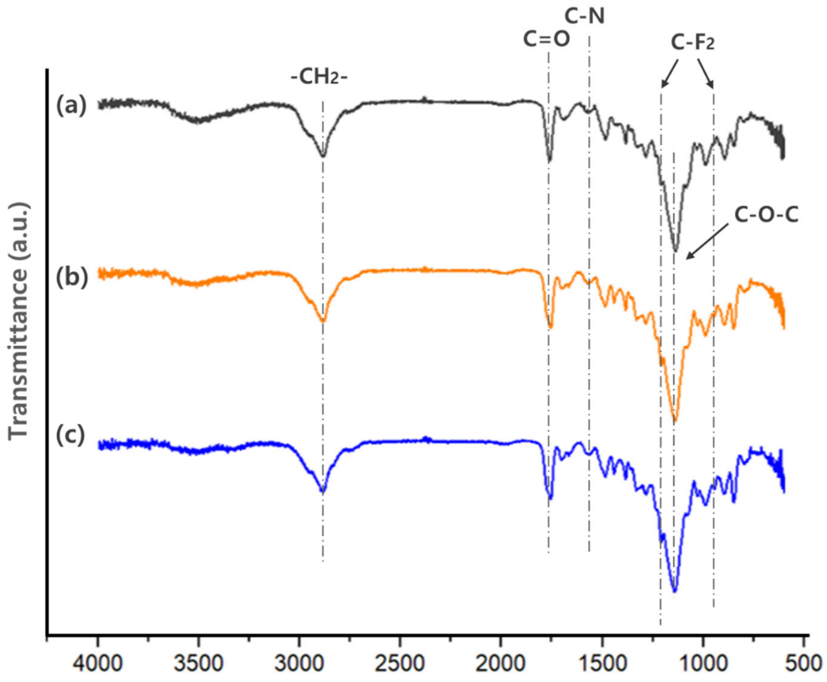

Figure S5. FT-IR spectra of (a) F20, (b) F30, and (c) F40.

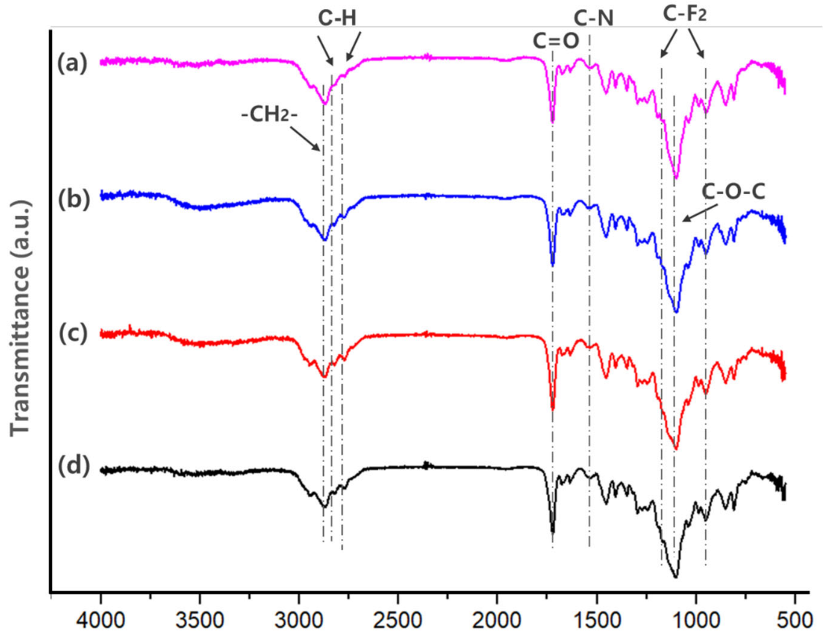

Figure S6. FT-IR spectra of (a) A20F10, (b) A30F10, (c) A40F10, (d) A40F20.

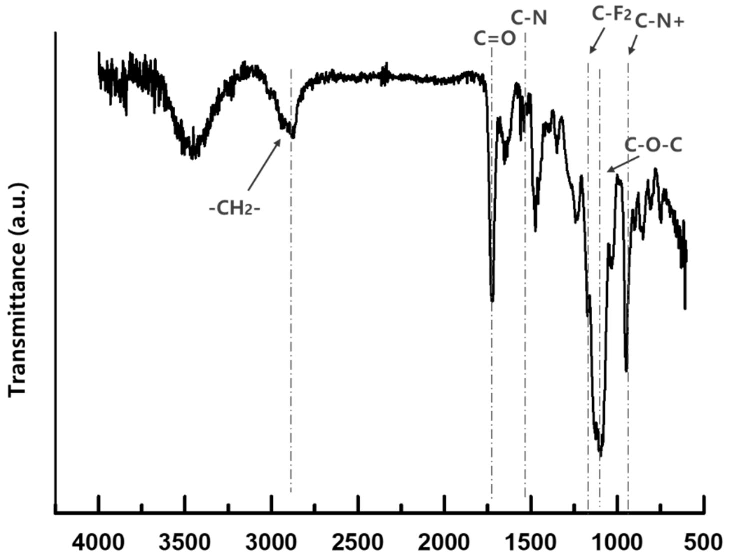

Figure S7. FT-IR spectra of A40F10+.

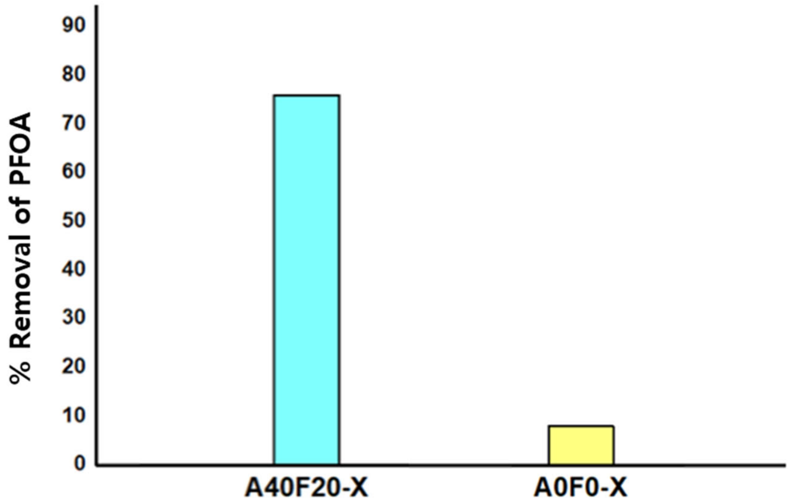

Figure S8. Removal efficiency of A40F20-X and A0F0-X.

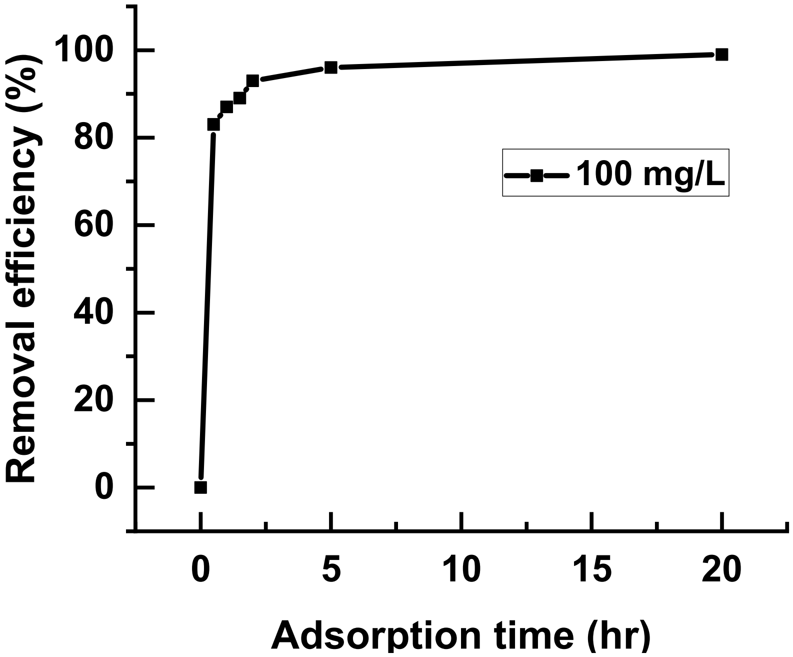

Figure S9. Adsorption kinetics of PFOA using A40F20+ hydrogel at 100 mg/L.

Table S1. Summary of estimated adsorption capacity.

Sample	Adsorption Capacity (mg/g)
A20F10	2.4
A30F10	2.6
A40F10	2.8
A40F20	3.4
A40F10+	4.0
A40F20+	4.0

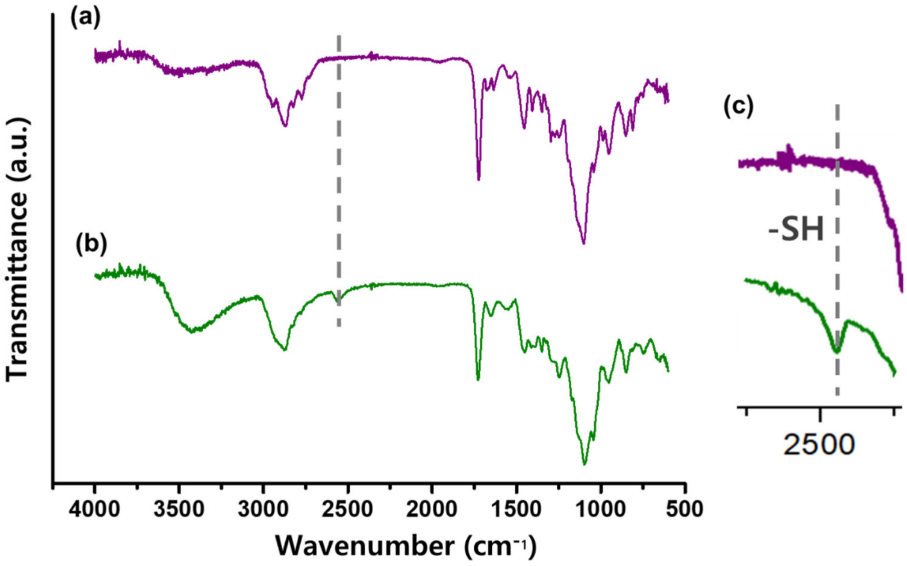

Figure S10. FT-IR spectra (a) before and (b) after DTT treatment. (c) Magnified spectra near 2460 cm^{-1} showing the presence of -SH peak after DTT treatment.

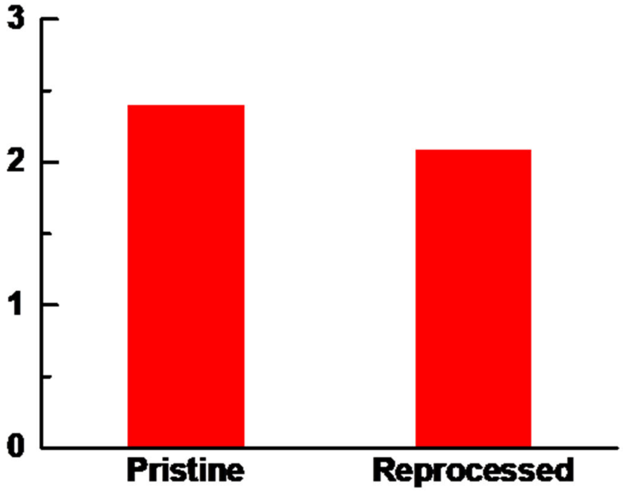

Figure S11. Adsorption capacity of pristine hydrogel and reprocessed hydrogel.

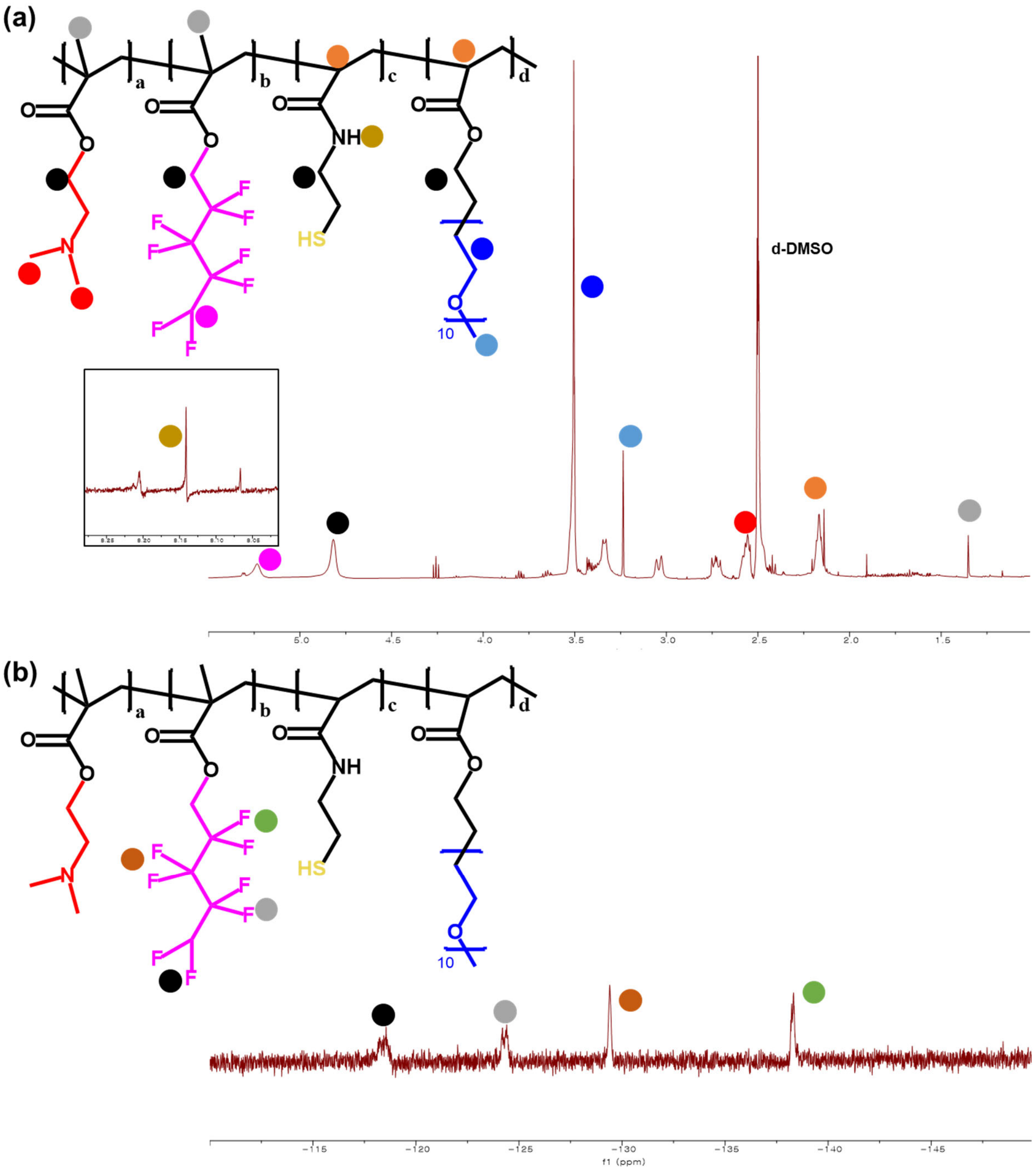

Figure S12. (a) ^{1}H and (b) ^{19}F NMR spectra of the polymer after cleavage of disulfide bond.

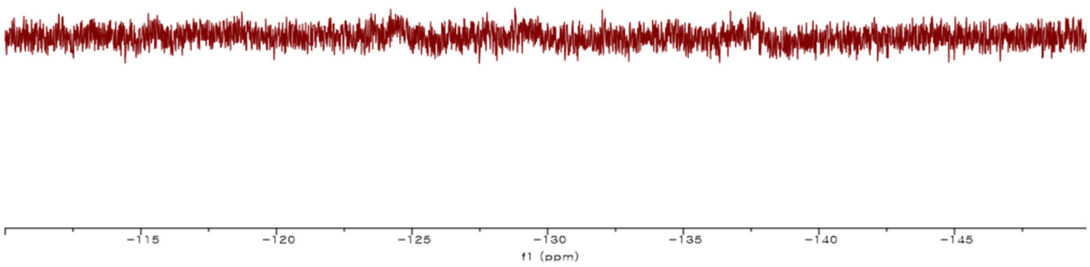

Figure S13. ^{19}F NMR of the micelle in D_2O after the self-assembly, showing disappearance of fluorine signals to reflect that the OFPMA groups constitute the core.
